# Assessment of treatment impact on lymphatic filariasis in 13 districts of Benin: progress toward elimination in nine districts despite persistence of transmission in some areas

**DOI:** 10.1186/s13071-019-3525-5

**Published:** 2019-05-30

**Authors:** Pelagie M. Boko-Collins, Aurore Ogouyemi-Hounto, Elvire G. Adjinacou-Badou, Laurinda Gbaguidi-Saizonou, Nissou Ines Dossa, Aboudou Dare, Moudachirou Ibikounle, Kathryn L. Zoerhoff, Daniel A. Cohn, Wilfrid Batcho

**Affiliations:** 1 National Control Program of Communicable Diseases, Ministry of Health of Benin, 01-BP-882, Cotonou, Benin; 2 Faculty of Health Sciences, University of Abomey-Calavi, 01BP 526, Cotonou, Benin; 3 RTI International, Cotonou, Benin; 4 Department of Zoology, Faculty of Sciences and Techniques, University of Abomey-Calavi, 01 BP 526, Cotonou, Benin; 5 RTI International, 701 13th Street NW, Suite 750, Washington, DC 20005 USA

**Keywords:** Active transmission, Benin, Mass drug administration, Lymphatic filariasis

## Abstract

**Background:**

Lymphatic filariasis (LF) is still a public health burden in many developing countries. In Benin, a West African country, at least 6.6 million people are at risk for LF. With the goal of eliminating LF by 2020, mass drug administration (MDA) has been scaled-up during the last decade. Currently, 23 districts are believed to have eliminated LF as a public health problem, and 25 other districts are still under treatment. In this study we report the results of the first transmission assessment survey of LF (TAS1) in 13 districts from the second group, which have received at least six rounds of MDA with albendazole and ivermectin.

**Methods:**

The 13 districts were grouped into six evaluation units (EU). In each EU, 30 schools randomly selected by survey sample builder (SSB) software were surveyed. Children aged six and seven were sampled in schools and for each child the Alere™ Filariasis Test Strip test was carried out using finger-prick blood to detect the circulating filarial antigen from *Wuchereria bancrofti*.

**Results:**

Overall, 9381 children were sampled in 191 schools from the six EU with 47.6% of the children aged six years and 52.4% aged seven years. Five EU passed the assessment, with no positive cases identified. The EU of Ouinhi which grouped the districts of Ouinhi, Cove, Za-Kpota and Zagnanado failed, with 47 positive cases. These cases were clustered in the districts of Ouinhi (*n* = 20), Za-Kpota (*n* = 11) and Zagnanado (*n* = 16). No cases were found in the district of Cove.

**Conclusions:**

The findings of our study indicate that Benin has made important progress towards elimination in most districts evaluated. However, this study also shows that transmission of LF is ongoing in the EU of Ouinhi, part of the Zou department. The MDA strategy needs to be strengthened in order to control the human reservoir of infection in these districts.

**Electronic supplementary material:**

The online version of this article (10.1186/s13071-019-3525-5) contains supplementary material, which is available to authorized users.

## Background

Lymphatic filariasis (LF) is a vector-borne parasitic disease endemic in several countries in Africa, Asia and the Americas. Currently 856 million people in 52 countries around the world live in areas where they are at risk of LF of which 499.4 million no longer require treatment to prevent the disease [1]. It is estimated that 91% of LF cases are caused by *Wuchereria bancrofti* while *Brugia malayi* and *Brugia timori* infections account for the remaining 9% [2]. As with many neglected tropical diseases, LF is common in disadvantaged communities and in Africa it is a significant public health burden [3]. LF is the second most common vector-borne disease after malaria [4] and a significant cause of long-term disability and mental illness [5–7]. In general, the majority of infected people do not present any visible symptoms at the early stage of the disease development even though they have been subjected to numerous cumulative infective bites which will lead to the development of LF-disability. In some cases, infected people may only briefly suffer from the debilitating effect of acute filarial episodes [8]. Hence, LF is likely to be underdiagnosed, especially in impoverished communities where health facilities have limited resources to detect the infection, leading patients to remain undiagnosed until the late stage when the disability caused by this nematode is noticeable.

In Benin, West Africa, approximately 6.6 million people are at risk of LF from *W. bancrofti*. As in many West African countries, the parasite is mainly transmitted by *Anopheles* mosquitoes [9–11]. The number of morbidity cases related to this disease has yet to be estimated in Benin, although efforts have being made to collect this information during mass drug administration (MDA). MDA has proven to be an effective strategy to eliminate LF [12, 13] and is the main elimination strategy adopted by Benin. Each year, treatment with the combination of ivermectin and albendazole is provided free of charge to all residents who are at least five years of age and live in endemic districts, excluding pregnant women, lactating women in the first week after birth and severely ill residents. Due to limited resources, treatments against LF were sporadic at the early control stage. With guidance from the Global Programme for the Elimination of Lymphatic Filariasis and the World Health Organization (WHO), and implementation support from a variety of partners, MDA has been scaled up and conducted at regular intervals in all endemic districts with the national goal of eliminating LF by 2020, in keeping with the global goal [14].

Initially, the mapping of LF indicated 50 endemic districts in 2000. In 2016, a remapping of Cotonou and Porto Novo, the two main urban settings of the country, were considered no longer endemic for LF bringing the total number of endemic districts from 50 to 48. Prior to the present study, 23 endemic districts received sufficient rounds of MDA and conducted TAS, which showed that prevalence has been lowered to a level at which MDA can be stopped. There are currently 25 other districts that are still under treatment against LF. Between 2013 and 2016, nocturnal microfilaremia assessments were conducted in 13 endemic districts of the 25 districts under treatment, all of which found less than 1% microfilaremia and were therefore eligible for their first transmission assessment survey (TAS) as recommended by the WHO [15]. The study reported here was conducted in order to evaluate whether or not the 13 districts could discontinue MDA.

## Methods

### Study sites and sample size

**Fig. 1:**
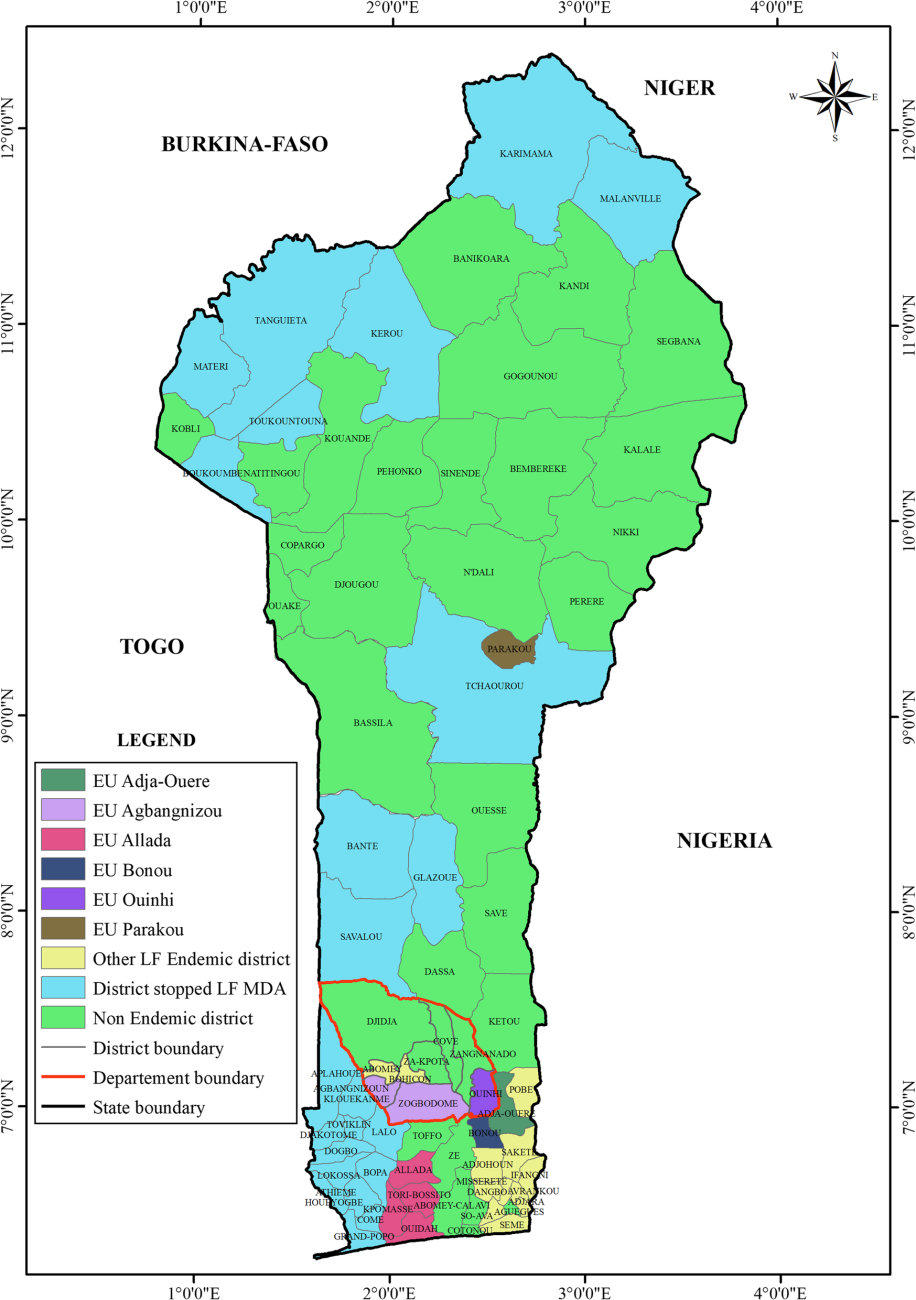
Map of Benin showing the districts where the transmission assessment survey was carried out

The TAS was conducted in schools in all 13 districts grouped into six evaluation units (Fig. 1). The evaluation unit (EU) of Allada included the districts of Allada, Ouidah, Kpomassè and Torri-Bossito; the EU of Ouinhi included the districts of Covè, Ouinhi, Za-Kpota and Zagnanado; the EU of Agbangnizoun included the districts of Zogbodomey and Agbangnizoun. The districts of Adja-Ouèrè, Bonou and Parakou each constituted an individual EU. All these endemic districts received several rounds of mass drug administration against LF and the most recent MDA was carried out in June 2017, nine months before the survey.

As the net school enrolment in each EU was above 75%, the sampling was carried out in schools following the WHO guidelines for TAS [15]. The list of schools surveyed and the sample size (Additional file 1: Tables S1–S6) in each district were generated using the TAS Survey Sample Builder (SSB) software [16]. This software is a tool specifically designed for TAS implementation by programme managers. The software takes into account the total population of the EU, the total number of children enrolled in the first and second grade of primary school, the total number of primary schools in the EU, the net enrolment rate and the LF vector, which is *Anopheles* in Benin [10]. The total number of children to sample varies according to the size of the EU.

### Training of surveyors

To ensure that the WHO guidelines were followed during data collection, teams of surveyors consisted of laboratory technicians and nurses, and supervisors were trained on the standard operating procedure to follow in the field. The training, led by the national team including medical doctors, a biologist and a statistician, focused on the modules of the WHO guide for TAS implementation and on the practical use of the Alere™ Filariasis Test Strip (FTS).

### Data collection

Only children aged six and seven who were enrolled in the selected schools were included in the survey. Children who were in the age range but have received treatment against LF within six months or who showed signs of illness (fever, etc.) were excluded from the survey.

The eligible children were randomly selected using one of the two randomization lists generated by the SSB software. Each selected child was assessed using an Alere™ Filariasis Test Strip which detects the specific antigen to *W. bancrofti*. A total of 75 μl of blood was collected using the capillary tube provided by the manufacturer. Each strip was identified by a unique code corresponding to each child before sampling. The blood sampling and the reading of the strips were carried out on the spot and in the presence of the children and the teachers and, on occasion, under the supervision of the parents or the representatives of the PTA. To ensure the validity of the tests carried out, the batches of FTS were tested with a positive control sample provided by the US-based Centers for Disease Control (CDC) prior to the survey.

Positive tests were repeated, as recommended by WHO, to confirm the result. When a positive test was repeated and was again positive then the result was confirmed as positive. When a positive test was repeated but was negative, the result was undetermined and was excluded from the sample.

### Data analysis

Data were recorded manually and cross checked. The data entered were analysed using SPSS software v.21 (IBM, Armonk, NY, USA). The critical threshold was determined by SSB software and varied by EU. It represents the maximum number of positive cases for which the EU still has a prevalence < 2% [15]. An EU “passes” the TAS if the number of confirmed positive cases is at or below the critical cut-off, at which point MDA can be discontinued. An EU “fails” the TAS if the number of confirmed positive cases is higher than the critical cut-off, prevalence > 2%, meaning that two more rounds of MDA must be implemented before reassessment. The Fisherʼs exact method of maximum likelihood and calculation of confidence intervals was used to calculate odds ratios by age in each EU.

## Results

### Sociodemographic characteristic of the populations surveyed

Overall 9381 children aged six and seven years were surveyed in the six evaluation units (EU). The characteristics of the children are summarized in Table 1. The sample was collected in 191 schools and consisted of 48.5% girls and 51.5% boys. Children aged six years represented 47.6% of the sample against 52.4% of children that were seven years of age. A total of 42.0% of the sampled children were in grade one and 57.1% in grade two. In the EU of Parakou, 1.7% of the eligible children were sampled in grade three.

**Table 1:** Sociodemographic characteristic of the populations surveyed

Evaluation unit	Adja-Ouèrè *n* (%)	Agbangnizoun *n* (%)	Allada *n* (%)	Bonou *n* (%)	Ouinhi *n* (%)	Parakou *n* (%)
Targeted sample size	1540	1552	1684	1380	1556	1556
Collected sample size	1571	1578	1710	1381	1572	1569
Targeted number of schools	30	30	30	30	30	30
Number of school surveyed	31	32	31	31	33	33
Children in first grade	713 (45.4)	698 (44.2)	693 (40.5)	643 (46.6)	673 (42.8)	576 (36.7)
Children in second grade	858 (54.6)	880 (55.8)	1017 (59.5)	738 (53.4)	899 (57.2)	966 (61.6)
Children in third grade	0 (0)	0 (0)	0 (0)	0 (0)	0 (0)	27 (1.7)
Six-year-old children	733 (46.7)	749 (47.5)	754 (44.1)	672 (48.7)	779 (49.6)	781 (49.8)
Seven-year-old children	838 (53.3)	829 (52.5)	956 (55.9)	709 (51.3)	793 (50.4)	788 (50.2)
Girls	737 (46.9)	803 (50.9)	813 (47.5)	671 (48.6)	764 (48.6)	7610 (48.5)
Boys	834 (53.1)	775 (49.1)	897 (52.5)	710 (51.4)	808 (51.4)	808 (51.5)

### Detection of *W. bancrofti* antigenemia in the evaluation units

**Fig. 2:**
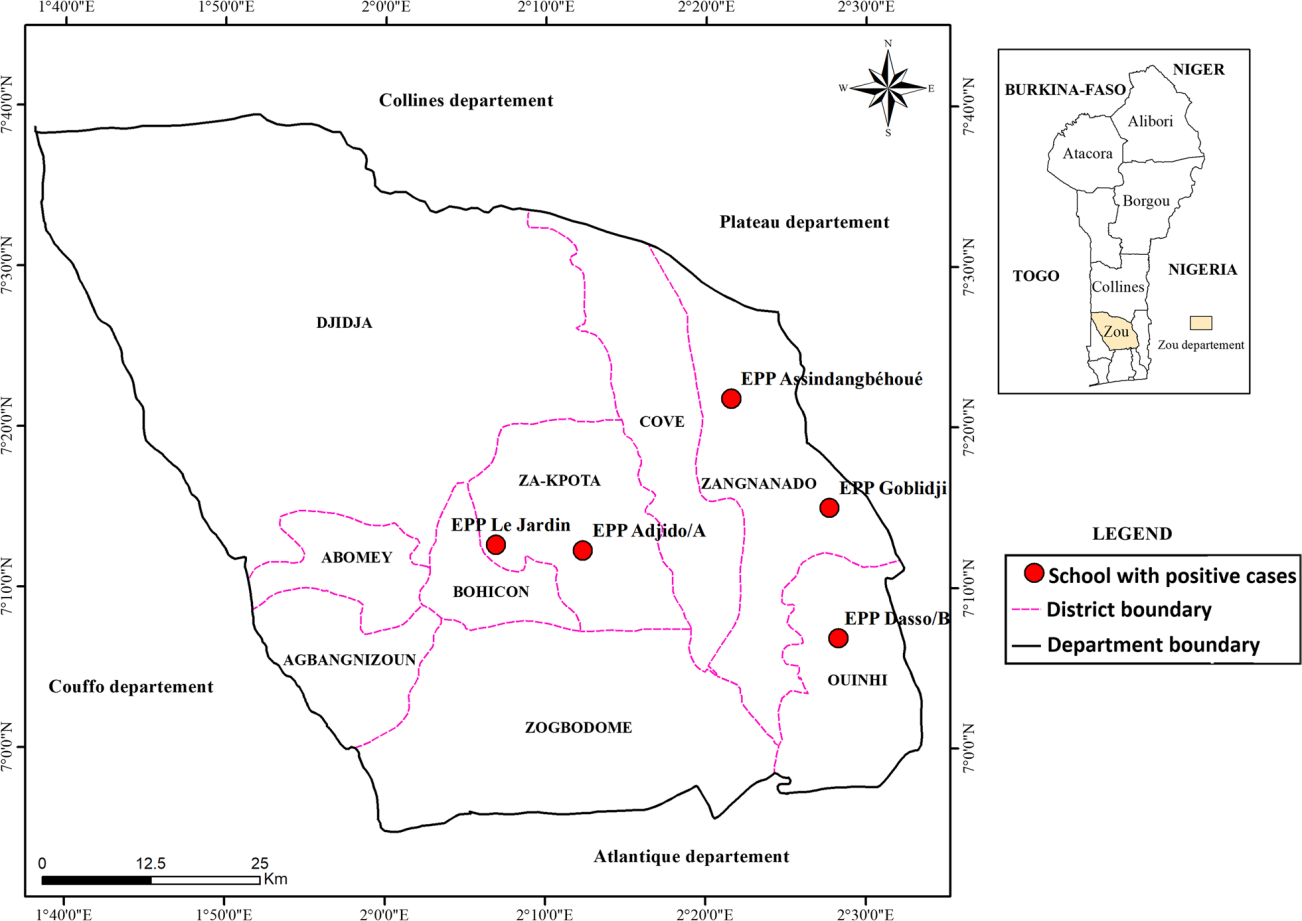
Map of the distribution of positive cases identified during the transmission assessment survey

Table 2 summarizes the number of positive cases of *W. bancrofti* antigenemia in the six EU. No cases of LF were identified in the EUs of Allada, Bonou, Adja-Ouèrè, Agbangnizoun and Parakou. In all five of these EUs, the number of positive cases was below the critical cut-off. In the EU of Ouinhi, 47 children were positive for *W. bancrofti* antigenemia. These positive cases were identified in three of the four districts that constituted the EU (Fig. 2). Among the 47 cases, 20 were in the district of Ouinhi, all in a single school; 16 were in the district of Zagnanado, including 12 in a single school in Baname sub-district and four in a single school in Don-Tan sub-district; and the remaining 11 cases were identified in the district of Za-Kpota, including six in a school in Za-Kpota sub-district and five in a school in Kpozoun sub-district. In addition, across the Ouinhi EU, a higher percentage of the infected children were aged seven-years (70%) than six-years (30%), of which 44.7% were girls and 55.3% were boys. The results also show that seven-year olds are twice as likely as their six-year old peers to be infected (OR = 2.4, 95% CI: 1.2–4.8, *P* = 0.007)

**Table 2:** Number of positive cases of *W. bancrofti* antigenemia in the six evaluation units

Evaluation unit	Adja-Ouèrè	Agbangnizoun	Allada	Bonou	Ouinhi	Parakou
Sample size	1571	1578	1710	1381	1572	1569
Critical cut-off	18	20	20	18	20	20
No. of positive samples on repeat test	0	0	0	0	47	0
No. of negative samples	1571	1578	1709^a^	1380^b^	1525	1569

^a^One sample was invalidated following the standard operating procedure recommended by the FTS fabricant (the sample first tested positive but negative after it was repeated)

^b^A child refused to provide a blood sample after he had been registered

## Discussion

In nine of the 13 districts assessed, LF prevalence has significantly been lowered compared to the baseline prevalence, as no positive cases were identified in the districts of Bonou, Adja-Ouèrè, Agbangnizoun, Zogbodomey, Parakou, Allada, Kpomassè, Ouidah and Tori-Bossito. The baseline prevalence obtained during the mapping in year 2000 is summarised in Table 3. The EU of Ouinhi showed transmission with positive cases in three (Za-Kpota, Zagnanado and Ouinhi) out of four districts. That EU consequently failed TAS1, in spite of 12 consecutive treatments with sufficient (≥ 65%) coverage of the total population reported to the National Control Programme of Communicable Diseases (NCPCD) (Additional file 2: Table S7).

**Table 3:** Baseline prevalence of the districts surveyed during TAS 1

District	Sample size	No. of LF cases	Prevalence (%)
Adja-Ouèrè (Adja-Ouèrè and Pobè^a^)	80	10	12.5
Allada (Allada and Tori-Bossito)	81	2	1.2
Ouidah (Kpomassè and Ouidah)	80	1	1.3
Agbangninzou (Agbangninzou, Abomey^a^ and Bohicon^a^)	61	1	1.6
Bonou (Bonou and Adjohoun^a^)	82	9	11.0
Ouinhi (Covè, Zagnanado and Ouinhi)	81	1	1.2
Parakou (Parakou)	84	2	2.5
Za-Kpota (Zogbodomey and Za-Kpota)	80	1	1.3

^a^These districts were not eligible for TAS 1 in 2018. For this assessment, eligible districts were therefore reorganised into EU following the WHO guideline

Give that the required duration of MDA is based on the estimated reproductive lifespan of the adult worm, at least five rounds of MDA with a minimum coverage of 65% of the total population is considered to be adequate in order to reduce microfilaremiae to a level at which transmission will end without further interventions [17]. However, similar trends have been observed in Ghana with a persistence of area transmission following 11 rounds of MDA [18]. Considering that the children sampled were born in and lived in their respective villages where they were sampled, they were born after at least four rounds of MDA against LF had been administered and thus should not have been exposed to infective bites. This suggests that the MDA coverage reported to the national level might not be credible; unfortunately, no coverage survey had been conducted in these EU during the treatment years. Therefore the NCPCD conducted a TAS failure investigation in the three districts with positives cases (Za-Kpota, Zagnanado and Ouinhi) in May 2018, using WHO’s Improving TAS Outcomes Checklists for Programme Managers [19].

Findings of the investigation included slight differences between reported and observed data in some villages, inconsistent adherence to directly-observed treatment (DOT), treatment with ivermectin alone when albendazole was out of stock, and prolonged absences of residents in some sub-districts. In the case of TAS failure, the WHO recommends at least two more years of MDA with sufficient coverage [15]. Recommendations from the investigation were to strengthen LF MDA in the entire EU by ensuring that health zone, district and sub-district personnel all perform supportive supervision; by training and supervising community drug distributors (CDD) to ensure DOT; by ensuring that nurses double-check the data reported by CDDs; and finally by ensuring that residents who are away during MDA are treated upon their return. The importance of the systematic application of DOT was noted in a prior review of determinants for success [20].

In 2013, sentinel site surveys (pre-TAS) conducted in the districts of Agbangnizoun, Ouinhi, Zagnanado, Za-Kpota, and Zogbodomey showed a prevalences of 1.17%, 0.38%, 0.52%, 1.98% and 0%, respectively (Benin pre-TAS Report 2013, unpublished data), suggesting the existence of ongoing transmission in these areas. The existence of such spots might not lead to a resurgence of LF [21, 22], although in Benin their distribution in these districts and throughout the ecological area appear to have worsened.

In this EU, there was a significant difference in the prevalence of LF between the six-year-old and seven-year-old children, which suggests that the latter have received an accumulation of infective bites over the years confirming that the prevalence of the disease would gradually increase with age [23]. Previous studies which had reported clustering of LF infected children similar to our study have indicated a variety of environmental factors enabling exposure to infective mosquito bites. Even though the coverage of long-lasting insecticide-treated nets (LLINs) in the department of Zou was close to 90% as of late 2011, four months after a LLIN distribution campaign [24], the difference of exposure between the age groups could be due to the fact that children under five are generally prioritised for bednet use in poor communities, consequently exposing older members of the family to infective bites. To ensure LF elimination in these districts, the NCPCD will associate the MDA strategy with the sensitization of the communities towards vector control adoption, specifically LLIN use.

Although our study was not specifically designed for this purpose, the results have also confirmed that LF is a disease acquired during childhood [25–27]. Due to the complexity of this disease which generally is almost silent in the early stage, untreated cases of *W. bancrofti* in the community constitute a reservoir of the disease. In households which do not receive the annual MDA, children are likely to be exposed to the infective bites. It is therefore imperative to emphasize these aspects during MDA sensitization to help the community understand that the drugs will not only help decrease the transmission of LF but will prevent the development of morbidity in later life [26].

## Conclusions

The NCPCD has set LF elimination target for 2020 and the absence of positive cases in nine of the 13 districts assessed indicate that Benin has succeeded in further reducing LF transmission. Benin now has a total of 32 districts which will no longer require MDA out of the 48 endemic districts. Nevertheless, the MDA strategy needs to be improved in the remaining districts in order to stop the spread of transmission spots, perhaps along with higher coverage of ITN use, and to control existent reservoir of LF in the entire country.

## Additional files

**Additional file 1: Table S1.** List of schools surveyed and sample size in the evaluation unit of Adja-Ouèrè. **Table S2.** List of schools surveyed and sample size in the evaluation unit of Bonou. **Table S3.** List of schools surveyed and sample size in the evaluation unit of Allada. **Table S4.** List of schools surveyed and sample size in the evaluation unit of Agbangnizoun. **Table S5.** List of schools surveyed and sample size in the evaluation unit of Ouinhi. **Table S6.** List of schools surveyed and sample size in the evaluation unit of Parakou.

**Additional file 2: Table S7.** Reported mass drug administration coverage in the surveyed districts since 2005.

## Data Availability

The datasets generated and analysed during the present study are not publicly available due to the policy of the Ministry of Health of Benin but are available from the corresponding author upon reasonable request.

## Abbreviations

LF: lymphatic filariasis
MDA: mass drug administration
TAS: transmission assessment survey
EU: evaluation unit
SSB: survey sample builder
USAID: United States Agency for International Development
RTI: Research Triangle Institute
MDP: Mectizan Donation Programme
WHO: World Health Organization
PTA: Parents and Teachers Associations
FTS: filariasis test strip
CDC: Centers for Disease Control and Prevention
NCPCD: National Control Programme of Communicable Diseases
DOT: directly-observed treatment
CDD: Community Drug Distributors
LLIN: long-lasting insecticide-treated nets
ITN: insecticide-treated nets
