# Supplementary material for: Assessment of treatment impact on lymphatic filariasis in 13 districts of Benin: progress toward elimination in nine districts despite persistence of transmission in some areas
Source: Parasit Vectors. 2019 May 30;12:276. doi: 10.1186/s13071-019-3525-5 (PMC6543600; doi:10.1186/s13071-019-3525-5)
Supplement: Supplementary file 1 — Additional file 1: Table S1. List of schools surveyed and sample size in the evaluation unit of Adja-Ouèrè. Table S2. List of schools surveyed and sample size in the evaluation unit of Bonou. Table S3. List of schools surveyed and sample size in the evaluation unit of Allada. Table S4. List of schools surveyed and sample size in the evaluation unit of Agbangnizoun. Table S5. List of schools surveyed and sample size in the evaluation unit of Ouinhi. Table S6. List of schools surveyed and sample size in the evaluation unit of Parakou. [file 13071_2019_3525_MOESM1_ESM.docx]

**Additional file 1: Table S1.** List of schools surveyed and sample size in the evaluation unit of Adja-Ouèrè

| N° | District | Sub-districts | School | Targeted Sample size |
| --- | --- | --- | --- | --- |
| 1 | ADJA-OUERE | ADJA-OUERE | EPP ADJA-OUERE-2/A | 52 |
| 2 | ADJA-OUERE | ADJA-OUERE | EPP ADJA-OUERE-3/B | 52 |
| 3 | ADJA-OUERE | ADJA-OUERE | EPP ATAKE-EKERE | 52 |
| 4 | ADJA-OUERE | ADJA-OUERE | EPP GBAHOUETE-TROBOSSI | 52 |
| 5 | ADJA-OUERE | ADJA-OUERE | EPP IGOÏSSI | 52 |
| 6 | ADJA-OUERE | ADJA-OUERE | EPP KADJOLA-TOFFO | 52 |
| 7 | ADJA-OUERE | ADJA-OUERE | EPP OKOFFIN | 52 |
| 8 | ADJA-OUERE | IKPINLE | EPP SAINT AUGUSTIN | 52 |
| 9 | ADJA-OUERE | IKPINLE | EPP SAINT MICHEL D'IKPINLE | 52 |
| 10 | ADJA-OUERE | IKPINLE | EPP SAINTE MARIE DES ANGES | 52 |
| 11 | ADJA-OUERE | IKPINLE | EPP ITA BOLARINWA/A | 52 |
| 12 | ADJA-OUERE | IKPINLE | EPP OKE-OWO | 52 |
| 13 | ADJA-OUERE | KPOULOU | EPP IGBO-AÏDIN | 52 |
| 14 | ADJA-OUERE | KPOULOU | EPP KPOULOU | 52 |
| 15 | ADJA-OUERE | MASSE | EPP LA JOIE DIVINE | 52 |
| 16 | ADJA-OUERE | MASSE | EPP ADJODA | 52 |
| 17 | ADJA-OUERE | MASSE | EPP IGBO IKOKO | 52 |
| 18 | ADJA-OUERE | MASSE | EPP ITCHOUGBO | 52 |
| 19 | ADJA-OUERE | MASSE | EPP MOWOBANI | 52 |
| 20 | ADJA-OUERE | MASSE | EPP OKE-OGNIBO | 52 |
| 21 | ADJA-OUERE | MASSE | EPP OLOROUN-SHOGO | 52 |
| 22 | ADJA-OUERE | OKO-AKARE | EPP ADJELEMIDE | 52 |
| 23 | ADJA-OUERE | OKO-AKARE | EPP SAINT ALPHONSE | 52 |
| 24 | ADJA-OUERE | OKO-AKARE | EPP KOKOROKEHOUN/B | 52 |
| 25 | ADJA-OUERE | OKO-AKARE | EPP LE DESIR | 52 |
| 26 | ADJA-OUERE | TATONNOUKON | EPP DJIDAGBA/A | 52 |
| 27 | ADJA-OUERE | TATONNOUKON | EPP IGBO-ARIN | 52 |
| 28 | ADJA-OUERE | TATONNOUKON | EPP LOGOU | 52 |
| 29 | ADJA-OUERE | TATONNOUKON | EPP LOHOUNGBODJE | 52 |
| 30 | ADJA-OUERE | TATONNOUKON | EPP TATONNONKON/B | 52 |

**Additional file 1: Table S2.** List of schools surveyed and sample size in the evaluation unit of Bonou

| N° | District | Sub-districts | School | Targeted Sample size |
| --- | --- | --- | --- | --- |
| 1 | BONOU | AFFAME | EPP SAINT ESPRIT | 46 |
| 2 | BONOU | AFFAME | EPP AFFAME-ABEOKOUTA | 46 |
| 3 | BONOU | AFFAME | EPP AGBOSSO-KOTA 1 | 46 |
| 4 | BONOU | AFFAME | EPP AGBOSSO-WOVIME/B | 46 |
| 5 | BONOU | AFFAME | EPP AGBOSSO-WOVIME/C | 46 |
| 6 | BONOU | ATCHONSA | EPP AGONHOUI | 46 |
| 7 | BONOU | ATCHONSA | EPP AGONKON/A | 46 |
| 8 | BONOU | ATCHONSA | EPP ATCHONSA/A | 46 |
| 9 | BONOU | ATCHONSA | EPP ATCHONSA/B | 46 |
| 10 | BONOU | ATCHONSA | EPP DOGBA | 46 |
| 11 | BONOU | ATCHONSA | EPP GBOA/B | 46 |
| 12 | BONOU | ATCHONSA | EPP KPAKPASSA | 46 |
| 13 | BONOU | ATCHONSA | EPP WETE | 46 |
| 14 | BONOU | ATCHONSA | EPP ZOUNGBOME | 46 |
| 15 | BONOU | BONOU | EPP AGBONAN/B | 46 |
| 16 | BONOU | BONOU | EPP ATCHABITA (EX ATCHABITA/A) | 46 |
| 17 | BONOU | BONOU | EPP LA VOIX DES ANGES | 46 |
| 18 | BONOU | BONOU | EPP BONOU-CENTRE/B | 46 |
| 19 | BONOU | BONOU | EPP GENIE DE LA VALEE | 46 |
| 20 | BONOU | BONOU | EPP TOVO | 46 |
| 21 | BONOU | DAME-WOGON | EPP AHOUANZONME/A | 46 |
| 22 | BONOU | DAME-WOGON | EPP AHOUANZONME/C | 46 |
| 23 | BONOU | DAME-WOGON | EPP ASSROSSA | 46 |
| 24 | BONOU | DAME-WOGON | EPP DAME WOGON (EX DAME WOGON/A) | 46 |
| 25 | BONOU | DAME-WOGON | EPP GNANHOUI-ZOUNME | 46 |
| 26 | BONOU | HOUNVIGUE | EPP ADIDO/A | 46 |
| 27 | BONOU | HOUNVIGUE | EPP ALIGODO | 46 |
| 28 | BONOU | HOUNVIGUE | EPP ALLANKPON (EX ALLANKPON/A) | 46 |
| 29 | BONOU | HOUNVIGUE | EPP HOUNVIGUE (EX HOUNVIGUE/A) | 46 |
| 30 | BONOU | HOUNVIGUE | EPP KODEMIAN | 46 |

**Additional file 1: Table S3.** List of schools surveyed and sample size in the evaluation unit of Allada

| N° | District | Sub-districts | School | Targeted Sample size |
| --- | --- | --- | --- | --- |
| 1 | ALLADA | AGBANOU | EPP TOKPA-AVAGOUDO | 57 |
| 2 | ALLADA | AHOUANNONZOUN | EPP HETIN-WETO | 57 |
| 3 | ALLADA | ALLADA 1 | EPP DOGOUDO/A | 57 |
| 4 | ALLADA | ALLADA 1 | EPP DOGOUDO/B | 57 |
| 5 | ALLADA | ATTOGON | EPP SAINT CECILE D'ATTOGON | 57 |
| 6 | ALLADA | AYOU | EPP AYOU-LANMADJI | 57 |
| 7 | ALLADA | LON-AGONMEY | EPP TOGAZOUN | 57 |
| 8 | ALLADA | SEKOU | EPP LA FAVEUR DIVINE | 57 |
| 9 | ALLADA | TOGOUDO (ALLADA 2) | EPP DAGLETA ASSOGON (EX TOGOUDO-FANDJI) | 57 |
| 10 | KPOMASSE | AGANMALOME | EPP AGANMANLOME | 57 |
| 11 | KPOMASSE | AGONKANME | EPP ASSOGBENOU KPEVI | 57 |
| 12 | KPOMASSE | DEDOME | EPP TELOKOE-AHOUYA | 57 |
| 13 | KPOMASSE | DEKANME | EPP HOUEYOGBE | 57 |
| 14 | KPOMASSE | KPOMASSE | EPP HOUENOUSSOU | 57 |
| 15 | KPOMASSE | AGBANTO | EPP CATHOLIQUE SAINT FRANCOIS D'ASSISE D'AGBANTO | 57 |
| 16 | KPOMASSE | TOKPA-DOME | EPP LOKOGBO | 57 |
| 17 | OUIDAH | DJEGBADJI | EPP DJEGBADJI | 57 |
| 18 | OUIDAH | HOUAKPE-DAHO | EPP DJEGBAME | 57 |
| 19 | OUIDAH | OUIDAH 1 | EPP KA SOURCE DU SUCCES | 57 |
| 20 | OUIDAH | OUIDAH 2 | EPP LEBOU/B | 57 |
| 21 | OUIDAH | OUIDAH 4 | EPP KPASSE/B | 57 |
| 22 | OUIDAH | OUIDAH 2 | EPP CERCLE DES GENIES | 57 |
| 23 | OUIDAH | OUIDAH 2 | EPP LEBOU/B | 57 |
| 24 | OUIDAH | OUIDAH 4 | EPP KPASSE/B | 57 |
| 25 | TORI-BOSSITO | AVAME | EPP HLA | 57 |
| 26 | TORI-BOSSITO | AZOHOUE-CADA | EPP GBEDACONOU | 57 |
| 27 | TORI-BOSSITO | TORI-BOSSITO | EPP CATHOLIQUE CONSEPTION TORI GARE | 57 |
| 28 | TORI-BOSSITO | TORI-BOSSITO | EPP WANHO | 57 |
| 29 | TORI-BOSSITO | TORI-CADA | EPP LA GLOIRE DU SEIGNEUR | 57 |
| 30 | TORI-BOSSITO | TORI-GARE | EPP GBEHOSSA | 57 |

**Additional file 1: Table S4.** List of schools surveyed and sample size in the evaluation unit of Agbangnizoun

| N° | District | Sub-districts | School | Targeted Sample size |
| --- | --- | --- | --- | --- |
| 1 | AGBANGNIZOUN | ADANHONDJIGON | EPP AGBOZOUNDJI | 52 |
| 2 | AGBANGNIZOUN | ADINGNINGON | EPP ADINGNIGON/B | 52 |
| 3 | AGBANGNIZOUN | AGBANGNIZOUN | EPP ECOLE CATHOLIQUE SAINT PIERRE | 52 |
| 4 | AGBANGNIZOUN | AGBANGNIZOUN | EPP AKPEHO-SEME/B | 52 |
| 5 | AGBANGNIZOUN | KINTA | EPP KINTA CENTRE/B | 52 |
| 6 | AGBANGNIZOUN | LISSAZOUNME | EPP LES BOCATES | 52 |
| 7 | AGBANGNIZOUN | LISSAZOUNME | EPP ZOUNGBO-SEKIDJATO | 52 |
| 8 | AGBANGNIZOUN | SAHE | EPP LA LUMIERE DE SAHE | 52 |
| 9 | AGBANGNIZOUN | SINWE-KPOTA | EPP HOUNTO-QUARTIER | 52 |
| 10 | AGBANGNIZOUN | SINWE-LEGO | EPP SINWE-HOUNTO/A | 52 |
| 11 | AGBANGNIZOUN | TANVE | EPP DEKANME-TANVE/A | 52 |
| 12 | AGBANGNIZOUN | TANVE | EPP HOUALA-VEKPA | 52 |
| 13 | AGBANGNIZOUN | ZOUNGOUDO | EPP KPOTO/A (EX KPOTA-TOKPA/A) | 52 |
| 14 | ZOGBODOMEY | AKIZA | EPP DENOU-LISSEZIN/A | 52 |
| 15 | ZOGBODOMEY | AKIZA | EPP OUASSOUGON/A | 52 |
| 16 | ZOGBODOMEY | AVLAME | EPP ALLADAHO/A | 52 |
| 17 | ZOGBODOMEY | AVLAME | EPP KOTOKPA/B | 52 |
| 18 | ZOGBODOMEY | CANA 1 | EPP TEGBESSOU (EX CANA-DOGOUDO) | 52 |
| 19 | ZOGBODOMEY | CANA 2 | EPP CANA-GBANGNANME/C (EX CANA-GBANGNAN/B) | 52 |
| 20 | ZOGBODOMEY | DOME | EPP DOME/B | 52 |
| 21 | ZOGBODOMEY | KOUSSOUKPA | EPP KOUSSOUKPA/A | 52 |
| 22 | ZOGBODOMEY | KPOKISSA | EPP AHOUANDJITOME | 52 |
| 23 | ZOGBODOMEY | KPOKISSA | EPP KPOKISSA | 52 |
| 24 | ZOGBODOMEY | MASSI | EPP HON | 52 |
| 25 | ZOGBODOMEY | TANWE-HESSOU | EPP ADJOGON | 52 |
| 26 | ZOGBODOMEY | TANWE-HESSOU | EPP DON SONAFA | 52 |
| 27 | ZOGBODOMEY | TANWE-HESSOU | EPP ZINGA | 52 |
| 28 | ZOGBODOMEY | ZOGBODOMEY | EPP LES PIONNIERS DE L'EXCELLENCE | 52 |
| 29 | ZOGBODOMEY | ZOGBODOMEY | EPP ZOGBODOME/B | 52 |
| 30 | ZOGBODOMEY | ZOUKOU | EPP HLANHONOU/B | 52 |

**Additional file 1: Table S5.** List of schools surveyed and sample size in the evaluation unit of Ouinhi

| N° | District | Sub-districts | School | Targeted Sample size |
| --- | --- | --- | --- | --- |
| 1 | COVE | SOLLI | EPP SAINT AUGUSTIN | 52 |
| 2 | COVE | HOUEKO | EPP COVE/D | 52 |
| 3 | COVE | HOUIN-HOUNSO | EPP TOUE | 52 |
| 4 | COVE | NAOGON | EPP TOKPLEGBE/B | 52 |
| 5 | OUINHI | DASSO | EPP AGONKON | 52 |
| 6 | OUINHI | DASSO | EPP OUASSA | 52 |
| 7 | OUINHI | OUINHI | EPP COMPLEXE SCOLAIRE SAINT PAUL | 52 |
| 8 | OUINHI | SAGON | EPP ADAGBODJI | 52 |
| 9 | OUINHI | SAGON | EPP DOLIVI | 52 |
| 10 | OUINHI | SAGON | EPP TEZOUNME | 52 |
| 11 | ZAGNANADO | AGONLIN-HOUEGBO | EPP AGONLIN-HOUEGBO/B | 52 |
| 12 | ZAGNANADO | BANAME | EPP AISSIN-DANGBEHOUE | 52 |
| 13 | ZAGNANADO | BANAME | EPP DJIDONGO | 52 |
| 14 | ZAGNANADO | BANAME | EPP SOHOUE NDOKPO/A | 52 |
| 15 | ZAGNANADO | DON-TAN | EPP GOBLIDJI | 52 |
| 16 | ZAGNANADO | DOVI-CENTRE | EPP KIOBO | 52 |
| 17 | ZAGNANADO | KPEDEKPO | EPP IGBO-OLA | 52 |
| 18 | ZAGNANADO | ZAGNANADO | EPP LA JEUNESSE AMBITION | 52 |
| 19 | ZA-KPOTA | ALLAHE | EPP DOGBANLIN | 52 |
| 20 | ZA-KPOTA | ASSANLIN | EPP ASSANLIN/A | 52 |
| 21 | ZA-KPOTA | HOUNGOME | EPP GINKOLI | 52 |
| 22 | ZA-KPOTA | KPAKPAME | EPP AGBLAGUI | 52 |
| 23 | ZA-KPOTA | KPAKPAME | EPP TANGBE/A | 52 |
| 24 | ZA-KPOTA | KPOZOUN | EPP LE JARDIN | 52 |
| 25 | ZA-KPOTA | ZA-KPOTA | EPP ADJIDO/A | 52 |
| 26 | ZA-KPOTA | ZA-KPOTA | EPP LA PERSEVERENCE | 52 |
| 27 | ZA-KPOTA | ZA-KPOTA | EPP ZA-KPOTA CENTRE/C | 52 |
| 28 | ZA-KPOTA | ZA-TANTA | EPP YOHOUE | 52 |
| 29 | ZA-KPOTA | ZEKO | EPP DADAH GUEZO | 52 |
| 30 | ZA-KPOTA | ZEKO | EPP LA REFERENCE | 52 |

**Additional file 1: Table S6.** List of schools surveyed and sample size in the evaluation unit of Parakou

| N° | District | Sub-districts | School | Targeted Sample size |
| --- | --- | --- | --- | --- |
| 1 | PARAKOU | 1st Sub-district | EPP ABDOULAYE ISSA/C | 52 |
| 2 | PARAKOU | 1st Sub-district | EPP ALBARIKA/E | 52 |
| 3 | PARAKOU | 1st Sub-district | EPP BEYAROU | 52 |
| 4 | PARAKOU | 1st Sub-district | EPP CAMP-ADAGBE/C | 52 |
| 5 | PARAKOU | 1st Sub-district | EPP FORET (EX FORET/A) | 52 |
| 6 | PARAKOU | 1st Sub-district | EPP ENFANT EPANOUI | 52 |
| 7 | PARAKOU | 1st Sub-district | EPP MADINA/A | 52 |
| 8 | PARAKOU | 1st Sub-district | EPP N’KWEI-BUYA | 52 |
| 9 | PARAKOU | 1st Sub-district | EPP PARAKOU GARE/A | 52 |
| 10 | PARAKOU | 1st Sub-district | EPP LA VOLONTE DE DIEU | 52 |
| 11 | PARAKOU | 1st Sub-district | EPP SINAGOUROU/D | 52 |
| 12 | PARAKOU | 1st Sub-district | EPP TIBONA/B | 52 |
| 13 | PARAKOU | 1st Sub-district | EPP TITIROU/E | 52 |
| 14 | PARAKOU | 1st Sub-district | EPP TOUROU CENTRE/C | 52 |
| 15 | PARAKOU | 1st Sub-district | EPP WOBEKOU-GAH | 52 |
| 16 | PARAKOU | 2nd Sub-district | EPP ASSAGBINE BAKA/B | 52 |
| 17 | PARAKOU | 2nd Sub-district | EPP BANIKANNI-1/D | 52 |
| 18 | PARAKOU | 2nd Sub-district | EPP MON AVENIR A | 52 |
| 19 | PARAKOU | 2nd Sub-district | EPP BAOUERA/C | 52 |
| 20 | PARAKOU | 2nd Sub-district | EPP GRACE DIVINE | 52 |
| 21 | PARAKOU | 2nd Sub-district | EPP MONTAGNE/A | 52 |
| 22 | PARAKOU | 2nd Sub-district | EPP HIBISCU B | 52 |
| 23 | PARAKOU | 2nd Sub-district | EPP TRANZA-1/A | 52 |
| 24 | PARAKOU | 3rd Sub-district | EPP DOKPAROU-EST | 52 |
| 25 | PARAKOU | 3rd Sub-district | EPP LA ROSETTE DU NORD | 52 |
| 26 | PARAKOU | 3rd Sub-district | EPP KPASSA-GAMBOU | 52 |
| 27 | PARAKOU | 3rd Sub-district | EPP SNTN/C | 52 |
| 28 | PARAKOU | 3rd Sub-district | EPP WOKODOROU/A | 52 |
| 29 | PARAKOU | 3rd Sub-district | EPP WORE/B | 52 |
| 30 | PARAKOU | 3rd Sub-district | EPP SAINT JEAN EUDES | 52 |
