# Supplementary material for: Assessment of treatment impact on lymphatic filariasis in 13 districts of Benin: progress toward elimination in nine districts despite persistence of transmission in some areas
Source: Parasit Vectors. 2019 May 30;12:276. doi: 10.1186/s13071-019-3525-5 (PMC6543600; doi:10.1186/s13071-019-3525-5)
Supplement: Supplementary file 2 — Additional file 2: Table S7. Reported mass drug administration coverage in the surveyed districts since 2005. [file 13071_2019_3525_MOESM2_ESM.docx]

**Additional file 2: Table S7.** Reported mass drug administration coverage in the evaluation unit of Ouinhi since 2005.

|  |  | MDA coverage (%) by Year of treatment | | | | | | | | | | | | | |  | |
| --- | --- | --- | --- | --- | --- | --- | --- | --- | --- | --- | --- | --- | --- | --- | --- | --- | --- |
| Evaluation unit | **Districts** | **2005** | **2006** | **2007** | **2008** | **2009** | **2010** | **2011** | **2012** | **2013** | **2014** | **2015** | **2016** | **2017** | **# of MDA rounds** | |  |
| Adja-Ouèrè | Adja-Ouèrè | No MDA | No MDA | No MDA | No MDA | No MDA | No MDA | 83.32 | 74.99 | 86.18 | 88.14 | 81.23 | 83.67 | 84.25 | 7 | |  |
| Allada | Allada | No MDA | No MDA | No MDA | No MDA | No MDA | No MDA | No MDA | 76.23 | 101.22 | 79.83 | 79.78 | 80.68 | 85.86 | 6 | |  |
| Allada | Kpomassè | No MDA | No MDA | No MDA | No MDA | No MDA | No MDA | 98.24 | 83.53 | 99.76 | 84.47 | 85.44 | 79.30 | 83.24 | 7 | |  |
| Allada | Ouidah | No MDA | No MDA | No MDA | No MDA | No MDA | No MDA | 96.43 | 82.60 | 62.99 | 81.39 | 76.70 | 86.15 | 89.18 | 7 | |  |
| Allada | Torri-Bossito | No MDA | No MDA | No MDA | No MDA | No MDA | No MDA | 93.00 | 78.13 | 71.10 | 78.01 | 85.56 | 78.33 | 94.35 | 7 | |  |
| Agbangnizoun | Agbangnizoun | No MDA | 79.63 | 77.56 | 74.45 | 77.85 | 78.00 | 67.82 | 54.33 | 80.19 | 82.43 | 87.73 | 86.80 | 87.24 | 12 | |  |
| Agbangnizoun | Zogbodomey | No MDA | 80.39 | 90.58 | 82.74 | 86.48 | 82.73 | 82.58 | 72.72 | 82.65 | 80.69 | 82.14 | 85.10 | 83.43 | 12 | |  |
| Bonou | Bonou | No MDA | No MDA | No MDA | No MDA | No MDA | No MDA | 87.59 | 80.53 | 82.67 | 86.47 | 75.06 | 84.63 | 85.88 | 7 | |  |
| Ouinhi | Covè | 80.75 | No MDA | No MDA | No MDA | No MDA | No MDA | 88.96 | 99.88 | 81.13 | 82.47 | 90.18 | 85.33 | 90.32 | 8 | |  |
| Ouinhi | Ouinhi | No MDA | 81.31 | 81.96 | 78.73 | 79.87 | 89.62 | 96.81 | 98.26 | 68.66 | 84.99 | 88.35 | 81.77 | 85.46 | 12 | |  |
| Ouinhi | Zagnanado | No MDA | 87.32 | 83.46 | 89.89 | 82.11 | 92.66 | 98.28 | 98.14 | 99.71 | 77.19 | 90.77 | 85.89 | 87.61 | 12 | |  |
| Ouinhi | Za-Kpota | No MDA | 78.80 | 91.61 | 79.23 | 90.29 | 89.98 | 74.53 | 89.67 | 90.24 | 87.73 | 80.48 | 86.03 | 84.90 | 12 | |  |
| Parakou | Parakou | No MDA | No MDA | No MDA | No MDA | No MDA | No MDA | 88.83 | 80.41 | 213.73 | 77.18 | 86.00 | 88.18 | 91.20 | 7 | |  |

(%): Percentage; #: Number; MDA: Mass drug administration. Some of the coverages reported here are above 100%, indicating the challenges faced by NCPCD during MDA implementation.
